# Withholding parenteral nutrition for 1 week reduces ICU-acquired weakness

**DOI:** 10.1186/cc12190

**Published:** 2013-03-19

**Authors:** G Hermans, B Clerckx, T Vanhullebusch, F Bruyninckx, M Casaer, P Meersseman, D Mesotten, S Vancromphaut, P Wouters, R Gosselink, A Wilmer, G Van den Berghe

**Affiliations:** 1 UZ Leuven, Belgium

## Introduction

ICU-acquired weakness (ICUAW) is a frequent and important complication of critical illness [1]. A large randomized controlled trial (EPaNIC: clinicaltrials.gov: NCT00512122) [2] showed that withholding parenteral nutrition during the first week of ICU stay whereby tolerating substantial caloric deficit (late PN) accelerated recovery and shortened weaning time as compared with early parenteral substitution for deficient enteral feeding (early PN). We examined the impact of late PN, as compared with early PN, on incidence and recovery of ICUAW.

## Methods

A preplanned subanalysis of adult patients included in the EPaNIC trial. The study was performed between October 2008 and November 2010 and included those patients who required intensive care for ≥8 days as well as a computer-generated, admission category-matched, random sample of short-stay ICU patients, the latter to correct for possible bias evoked by earlier ICU discharge in one of the two study groups. Assessors blinded for treatment allocation evaluated muscle strength clinically three times weekly from awakening onward and performed nerve conduction studies and electromyography (NCS and EMG) weekly. The primary outcome was the incidence of ICUAW, diagnosed clinically by the Medical Research Council (MRC) sum score (<48/60) [3] at first evaluation. Secondary outcomes included ICUAW at worst and last MRC evaluation, recovery from ICUAW and incidence of abnormal findings on NCS and EMG. All analyses were performed on the total dataset and on a for-baseline characteristics propensity score-matched sample to correct for possible imbalances between the groups.

## Results

Clinical ICUAW evaluation was performed in 600 patients (matched *n = *558), electrophysiological testing in 730 (matched *n = *684). Late PN reduced the incidence of ICUAW at first evaluation from 43.1% to 34.4%, *P *= 0.03 (matched: early PN 41.6%, late PN 33.3%, *P *= 0.04). Significantly fewer patients in the late PN group developed weakness at any time during ICU stay (late PN 37.0%, early PN 46.4%, *P *= 0.02; matched: late PN 36.2%, early PN 45.2%, *P *= 0.03). ICUAW may have recovered faster with late PN than with early PN (*P *= 0.05, matched *P *= 0.06). Other outcomes were not different.

## Conclusion

As compared with early PN, late PN reduced the incidence of ICUAW and may have accelerated recovery thereof.
